# Sharpnel splinter in the common bile duct

**DOI:** 10.1093/omcr/omae088

**Published:** 2024-08-19

**Authors:** Ahmad Abbas, Faiz Al-theab

**Affiliations:** Department of Gastroenterology, Damascus Hospital, Ministry of Health, Damascus, Syria; Department of Gastroenterology, Damascus Hospital, Ministry of Health, Damascus, Syria

**Keywords:** common bile duct, foreign body, obstructive jaundice

## Abstract

Foreign bodies are a rare cause of obstructive jaundice. In this case report, we present the case of a 59-year-old male who presented with abdominal pain and obstructive jaundice, which was later found out to be caused by an impacted shrapnel splinter in the common bile duct 7 years after a combat injury. To our knowledge, this is the first documented case from Syria. This case report is a reminder that impacted foreign bodies should be considered as a potential cause of obstructive jaundice in patients with previous combat injury.

## Introduction

Foreign bodies in the biliary tract are a rare cause of obstructive jaundice. Therefore, they usually do not receive much attention. Such foreign bodies include: T- tubes, vessel clips, fish bones, bullets and sharpnel splinters. This report describes the case of a 59-year-old male who presented with obstructive jaundice caused by an impacted shrapnel in the common bile duct 7 years after the initial injury. To our best knowledge, this is the first documented case from syria. We also report on the findings on Endoscopic Retrograde Cholangiopancreatography (ERCP), discuss the management plan and point out to the idea that shrapnel splinters in the common bile duct should be taken into consideration as a differential diagnosis in patients who present with symptoms suggestive of obstructive jaundice and who have a history of combat injury.

## Case presentation

A 59-year-old male presented with a two-week history of jaundice and tenderness in the right upper quadrant (RUQ) of the abdomen and epigastrum, with radiation to the back. He did not suffer from fever or diarrhea, He sustained an abdominal injury during the conflict in Syria, and several sharpnel splinters settled in his abdomen, He has no medical history, and his surgical history extracting a military shrapnel from abdominal cavity, and no history of medication.

On physical examination, the patient had icterus, jaundice, and tenderness in the RUQ. A scar on the abdominal midline was also noted. Lab work was as shown on Table 1.

**Table 1:** Lab work

**Test**	**On admission**	**Discharge**	**Normal limits**	**Unit**
WBC	6500	9800	4500–10 500	/mm^3^
Hemoglobin	13.4	14	12–16	g/dl
Platelets	132	254	150–450	X ^1000^ mm^3^
Serum Amylase	1042		290	U/L
Urea	20		15–54	mg/dl
Creatinine	0.6		0.5–1.3	mg/dl
ALT	90		5–45	U/L
AST	53		8–40	U/L
Total Bilirubin	6.8		0.5–1.2	mg/dl
Direct Bilirubin	5.9		0–0.3	mg/dl
Alp	389		UP TO 290	U/L
Ggt	551		0–50	U/L
Pt/INR	49%/1.5	60%/1.3	70%	
Glucose	100	105	74–106	mg/dl
Crp	71.3	6		
Na	132	138	132–138	
K	3.3	4.1	3.5–5	
Ca19-9	632			U/milliliter

Abbreviations:

WBC: white blood cells.

ALT: alanine aminotransferase.

AST: aspartate aminotransferase.

Inr: international normalised ratio.

Alp: serum alkaline phosphatase.

Ca 19-9: carbohydrate antigen 19–9.

On abdominal ultrasonography, the gallbladder was normal, and the diameter of the intrahepatic ducts was within normal limits. However, the common bile duct was dilated (diameter = 100 millimeters). Based on the laboratory and radiological findings. Cholangiocarcinoma was suspected.

This dilation was presumed to be the probable cause of the patient’s symptoms. Therefore, we decided to perform a diagnostic (ERCP).

During the ERCP, we noted a filling defect in the proximal portion of the common bile duct (CBD) that was caused by an obstructive object.

**Figure 1:**
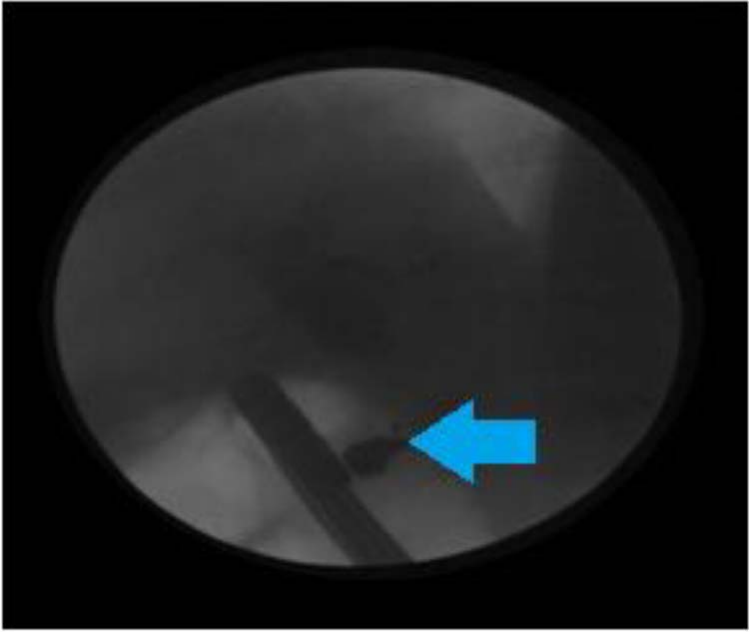
Endoscopic retrograde cholangiopancreatography showing a foreign body (The arrow) in the CBD before sphincterotomy.

The obstructive object roughly measured (6 × 7 millimeters) (Fig. 1).

**Figure 2:**
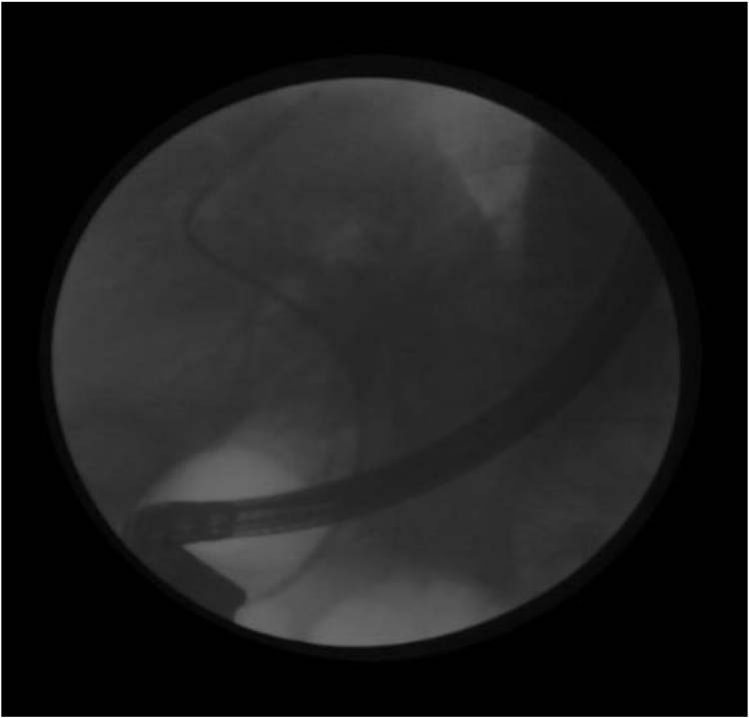
Endoscopic retrograde cholangiopancreatiography after extraction of the foreign body following sphincterotomy and placement of a plastic stent.

Endoscopic retrograde cholangiopancreatiography after extraction of the foreign body following sphincterotomy and placement of a plastic stent is shown in (Fig. 2).

**Figure 3:**
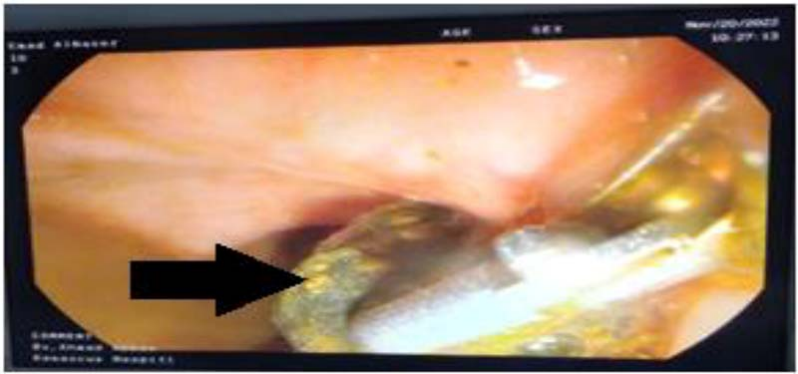
The shell splinter as a foreign body (black arrow) in the common bile duct as seen on endoscopic retrograde cholangiopancreatography.

We performed a sphincterotomy and extracted the obstructive object with a post-dilation balloon as shown in (Fig. 3).

**Figure 4:**
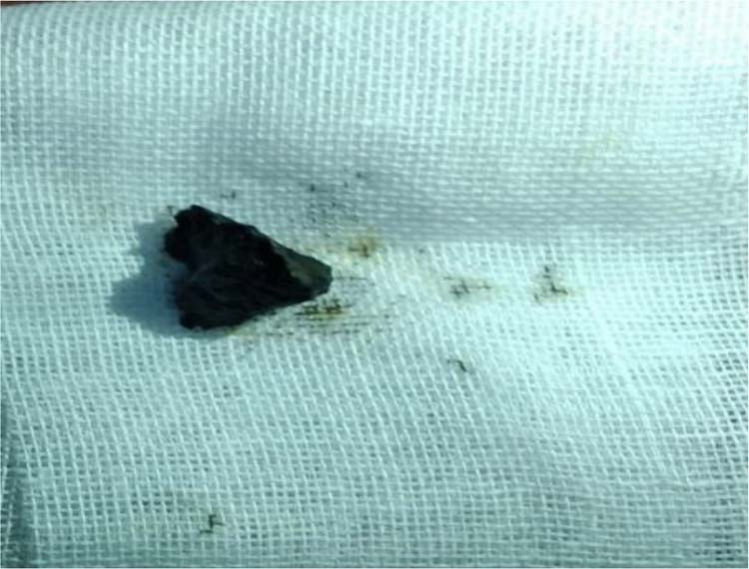
Splinter after extraction by upper Esophagogastroduodenoscopy (EGD).

A stone extraction basket was inserted using Esophagogastroduodenoscopy (EGD) as shown in (Fig. 4).

## Discussion

Biliary stones are not the only cause of obstructive jaundice. There are a variety of predisposing factors, such as narrowing of the anastomoses between the biliary tract and the duodenum after surgical interventions, malignant and benign strictures. Foreign bodies in the CBD are a rare cause. However, it should be considered as a cause, especially in people with a history of a combat injury, regardless of the initial entry point of the sharpnel splinters. The entrance point of the shrapnel splinters is not limited to the abdomen. There have been cases where the entry point was the thoracic cavity [1].

Although, obstructive jaundice is the most common symptom of foreign bodies in the CBD, symptoms may be limited to abdominal pain [2].

Chronic obstruction of the biliary tract may be complicated by sclerosing cholangitis [3].

We note from the cases shown in Table 2 that symptoms caused by foreign bodies in the CBD has a latency period ranging from months to years before they appear.

**Table 2:** Characteristics of the reported cases of missiles in the biliary tree from combat injury

**Reference**	**Positive symptoms and signs on admission**	**Type of foreign body**	**Age (yr)/gender**	**Original injury**	**Latent period**	**Site of migration**
Eghuchi [1]	Icterus, pruritus	Shrapnel splinter	60/M	Right thoracic cavity	36 yr	**CBD**
Kamona [2]	Abdominal pain	Bullet	14/M	Liver parenchyma	4 mo	**CBD**
Silvermann [3]	Icterus, fever, abdominal pain	Shrapnel	75/M	RUQ	Initial injury in World War 2	**CBD**
Mitchell [4]	Fever, weight loss, nausea, vomiting, abdominal pain icteric sclera	Shrapnel splinter	64/M	RUQ	44 yr	**CBD**
Hussain [5]	Jaundice, right hypochondrial pain, vomiting, fever, weakness, fatigue and weight loss	Bullet	26/M	Right lobe of the liver	9 yr	**CHD**
Klein [6]	Jaundice, abdominal pain	Shell splinter	44/M	Foramen of winslow	9 yr	**direct penetration to CBD**
Rescorla [7]	Abdominal pain in RUQ2, Scleral icterus	Bullet	8/F	Left lobe of the liver	22 mo	**CHD**
Krontiris [8]	Fever, jaundice, acholic stool, anorexia, malaise, and weakness, abdominal pain in RUQ	Cap of a bullet	36/M	Right lower thorax	13 yr	**CBD**
Gottschalk [9]	Abdominal pain and tenderness in RUQ, jaundice	Shell splinter	69/M	-	49 yr	**CBD**
Somi MH [10]	Jaundice, abdominal pain in RUQ	Shrapnel splinter	42/M	RUQ and right thorax	23 yr	**CBD**
Present case	Jaundice, pain in the RUQ (right upper quadrant) of the abdomen and epigastrum	Shrapnel splinter	59/M	Abdomen	7 yr	**CBD**

Foreign bodies in the CBD may sometimes present with symptoms indicative of malignancy such as weight loss [4, 5]. In our case, the patient had elevated CA 19-9 levels.

## Summary

Foreign bodies in the biliary tract that cause of obstructive jaundice miscellaneous, that a missed gauze in the abdominal cavity after surgery on abdominal cavity, Therefore, it is important to take the patient’s medical history carefully to determine the cause of obstructive jaundice.
